# Modulation of NBAS-Related Functions in the Early Response to SARS-CoV-2 Infection

**DOI:** 10.3390/ijms24032634

**Published:** 2023-01-30

**Authors:** Valentina Granata, Isabel Pagani, Emanuela Morenghi, Maria Lucia Schiavone, Alessandra Lezzi, Silvia Ghezzi, Elisa Vicenzi, Guido Poli, Cristina Sobacchi

**Affiliations:** 1 Milan Unit, National Research Council—Institute for Genetic and Biomedical Research, 20138 Milan, Italy; 2 Human Genome and Biomedical Technologies Unit, IRCCS Humanitas Research Hospital, 20089 Milan, Italy; 3 Viral Pathogenesis and Biosafety Group, San Raffaele Scientific Institute, 20132 Milan, Italy; 4 Biostatistics Unit, IRCCS Humanitas Research Hospital, 20089 Milan, Italy; 5 School of Medicine, Vita-Salute San Raffaele University, 20132 Milan, Italy

**Keywords:** SARS-CoV-2, Calu3, nonsense-mediated decay, retrograde transport, NBAS, inflammation

## Abstract

Upon infection, severe acute respiratory syndrome—coronavirus 2 (SARS-CoV-2) is predicted to interact with diverse cellular functions, such as the nonsense-mediated decay (NMD) pathway, as suggested by the identification of the core NMD factor upframeshift-1 (UPF1) in the SARS-CoV-2 interactome, and the retrograde transport from the Golgi to the endoplasmic reticulum (ER) through the endoplasmic reticulum–Golgi intermediate compartment (ERGIC), where coronavirus assembly occurs. Here, we investigated the expression and localization of the neuroblastoma-amplified sequence (NBAS) protein, a UPF1 partner for the NMD at the ER, participating also in retrograde transport, and of its functional partners, at early time points after SARS-CoV-2 infection of the human lung epithelial cell line Calu3. We found a significant decrease of DExH-Box Helicase 34 (*DHX34)*, suppressor with morphogenetic effect on genitalia 5 (*SMG5)*, and *SMG7* expression at 6 h post-infection, followed by a significant increase of these genes and also *UPF1* and *UPF2* at 9 h post-infection. Conversely, *NBAS* and other genes coding for NMD factors were not modulated. Known NMD substrates related to cell stress (Growth Arrest Specific 5, *GAS5;* transducin beta-like 2, *TBL2*; and DNA damage-inducible transcript 3, *DDIT3*) were increased in infected cells, possibly as a result of alterations in the NMD pathway and of a direct effect of the infection. We also found that the expression of unconventional SNARE in the ER 1, *USE1* (p31) and Zeste White 10 homolog, *ZW10*, partners of NBAS in the retrograde transport function, significantly increased over time in infected cells. Co-localization of NBAS and UPF1 proteins did not change within 24 h of infection nor did it differ in infected versus non-infected cells at 1 and 24 h after infection; similarly, the co-localization of NBAS and p31 proteins was not altered by infection in this short time frame. Finally, both NBAS and UPF1 were found to co-localize with SARS-CoV-2 S and N proteins. Overall, these data are preliminary evidence of an interaction between NBAS and NBAS-related functions and SARS-CoV-2 in infected cells, deserving further investigation.

## 1. Introduction

Coronaviruses are enveloped positive-sense single-stranded RNA viruses responsible for most of the seasonal mild respiratory tract infections normally present in humans, other mammalians, and avian species [1,2] and for more serious respiratory manifestations, such as those elicited by the severe acute respiratory syndrome coronavirus 2 (SARS-CoV-2) [3].

SARS-CoV-2 particles present a nucleocapsid containing the viral genomic RNA packaged by the nucleocapsid protein (N), surrounded by an envelope displaying different structural proteins, namely the envelope (E), membrane (M), and spike (S) proteins [4]. Upon entry [5,6], the viral genomic RNA is uncoated and translated, giving rise to two large polyproteins further processed into the individual nonstructural proteins (nsps) that form the viral replication and transcription complex. At the same time, extensive reorganization of cellular membranes generates the viral replication organelles consisting of characteristic perinuclear double-membrane vesicles, convoluted membranes, and small open double-membrane spherules [7], which overall constitute a protective microenvironment for viral genomic RNA replication and transcription of subgenomic mRNAs coding also for the viral structural proteins. These latter translocate into the endoplasmic reticulum (ER) and transition through the endoplasmic-reticulum-to-Golgi intermediate compartment (ERGIC), where new viral particles assemble and enter the exocytotic pathway for virion release and spreading of infection. Throughout its life cycle, the virus on the one hand takes advantage of host factors useful for sustaining infection [8] and on the other hijacks potentially harmful cell processes [9], such as nonsense-mediated decay (NMD).

NMD is an ubiquitous key surveillance mechanism aimed at preventing the accumulation of possibly damaging endogenous as well as exogenous proteins [10]. This pathway recognizes and degrades mRNAs bearing typical aberrant features, such as a premature translation-termination codon, an unusually long 3′-untranslated region, or upstream open reading frames; all features displayed also by viral RNAs. Several molecules participate in each step of the process. Among them, the key players are the ATP-dependent RNA helicase upframeshift-1 (UPF1) and the kinase suppressor with morphogenetic effect on genitalia 1 (SMG1), which, together with eukaryotic release factors 1 and 3 (eRF1 and eRF3), form the SMG-1-Upf1-eRF1-eRF3 complex at the stalled ribosome in the detection phase. Then, in the commitment phase, UPF2, anchored at the exon-junction complex through UPF3, interacts with UPF1, stimulating its phosphorylation by SMG1. Finally, in the degradation phase, phosphorylated UPF1 recruits SMG6 that has direct endonuclease activity, and/or the SMG5/SMG7 heterodimer, which, in turn, enrols decapping and deadenylating enzymes for exonucleolytic decay of the target molecule [4,9]. Viruses have developed different strategies to escape NMD, such as engaging host factors to their own advantage; of note, the SARS-CoV-2 N protein has been predicted to interact with the core NMD factor UPF1 [11].

In 2013, Longman and colleagues found two additional factors involved in the NMD pathway: DExH-Box Helicase 34 (DHX34) and neuroblastoma-amplified sequence (NBAS) proteins; indeed, the depletion of either of the two reduced the expression of core NMD factors [12]. Further evidence demonstrated that NBAS is a surveillance factor in ER-targeted NMD [13], and that it participates also in other functions, most importantly Golgi-to-ER retrograde traffic, as a key component of the syntaxin 18 complex [14], and in bulky cargo export from the ER orchestrated by the TANGO1 protein [15]. In the last ten years, *NBAS* has been attracting interest in the field of human pathology, owing to the spectrum of phenotypes associated with mutations in this gene [16,17,18,19,20].

**Figure 1:**
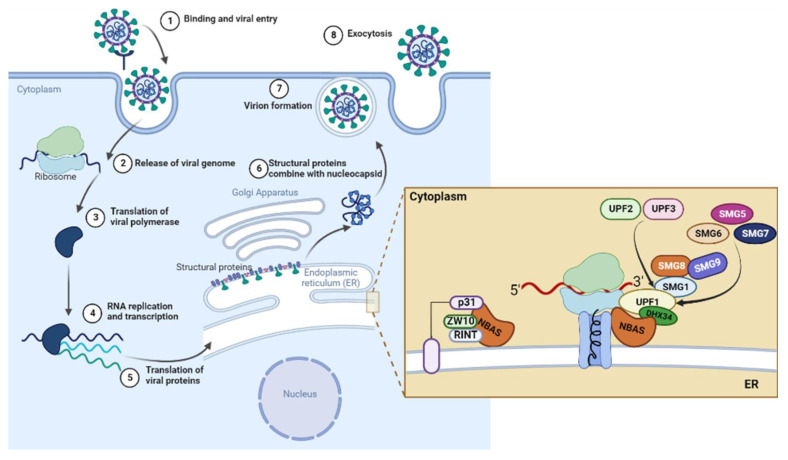
Schematic representation of SARS-CoV-2 infection and replication cycle in host cells. Zoom on cellular mechanisms involving NBAS protein herein investigated. The figure was created with BioRender.com (accessed on 12 December 2022).

In this overall scenario, we asked whether NBAS might be involved in the pathological mechanisms elicited by SARS-CoV-2 infection. To answer this question, we assessed NBAS expression and function in the NMD pathway and Golgi-to-ER transport, and key players of these cellular mechanisms, in SARS-CoV-2-infected Calu3 cells (Figure 1), which are an established in vitro model of infection relevant to human pathology. In our observation time frame, we found modest molecular changes likely allowing, on the one hand, cell survival and, on the other, virus replication.

## 2. Results

### 2.1. Establishment of the SARS-CoV-2 Infection Model in the Calu3 Cell Line

**Figure 2:**
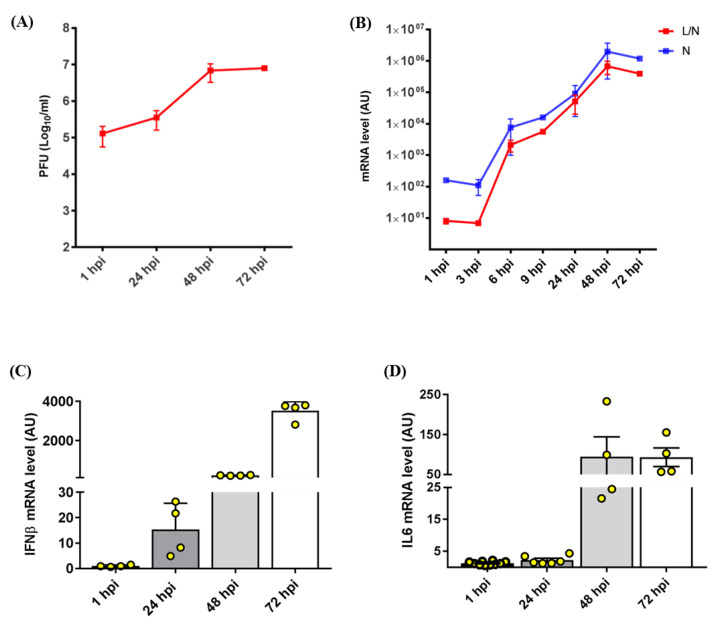
SARS-CoV-2 infection model in Calu3 cells. (**A**) Virus titer in SARS-CoV-2-infected Calu3 cells, expressed as plaque-forming units (PFU)/mL along time (hours post-infection, hpi). (**B**) Expression profile (arbitrary units, AU) of genomic (N) and subgenomic (L/N) viral sequences in SARS-CoV-2-infected Calu3 cells, assessed by qRT-PCR at different time points. The mean of two or five independent experiments ± standard error of the mean (SEM) is indicated for each time point. (**C**) Expression levels (arbitrary units, AU) of the inflammatory cytokines *IFN-β* and (**D**) *IL6* in infected versus non-infected Calu3 cells at different time points (hours) post-infection. Each bar represents the mean of two or five independent experiments ± SEM. Infected cells were compared with non-infected Calu3 cells for each time point. One-way ANOVA with the Bonferroni multiple comparison test.

The human lung epithelial cell line Calu3 was infected with a SARS-CoV-2 clinical isolate [21] at a multiplicity of infection of 3. Viral replication was quantified through an optimized plaque forming unit assay [22], at 1, 24, 48 and 72 h post-infection. We observed an increase of the virus titer over time (Figure 2A). Accordingly, the accumulation of genomic (N) and subgenomic (L/N) viral sequences exponentially increased between 1 and 24 h post-infection and remained persistently high at 72 h post-infection (Figure 2B). Overall, these results indicated a productive infection. Expression analysis of cellular genes, performed also at short time points (3, 6, and 9 h post-infection) showed that the expression of the inflammatory genes coding for IFN-β (interferon beta) and IL-6 (interleukin 6), involved in innate immune responses during viral infection, increased over time (Figure 2C,D), in parallel with the raise in viral load.

### 2.2. Gene Expression Analysis in SARS-CoV-2-Infected Calu3 Cells

#### 2.2.1. NBAS and NMD Key Factors

To assess whether NBAS and core NMD factors, including DHX34 and members of the UPF and SMG families, were affected in our SARS-CoV-2 infection model, we quantified their expression in the RNA isolated from infected and non-infected Calu3 cells at different time points, by quantitative RT-PCR. The NMD pathway is a multistep process, and the protein products of the investigated genes participate in different phases: initiation (DHX34, UPF1, UPF2, UPF3, SMG1, SMG8, and SMG9), commitment (UPF1 and SMG1), and degradation (SMG family members other than SMG1). On the other hand, NBAS fulfils an independent function, as it activates an ER-dedicated UPF1-dependent NMD pathway specifically regulating membrane-associated mRNAs [23].

**Figure 3:**
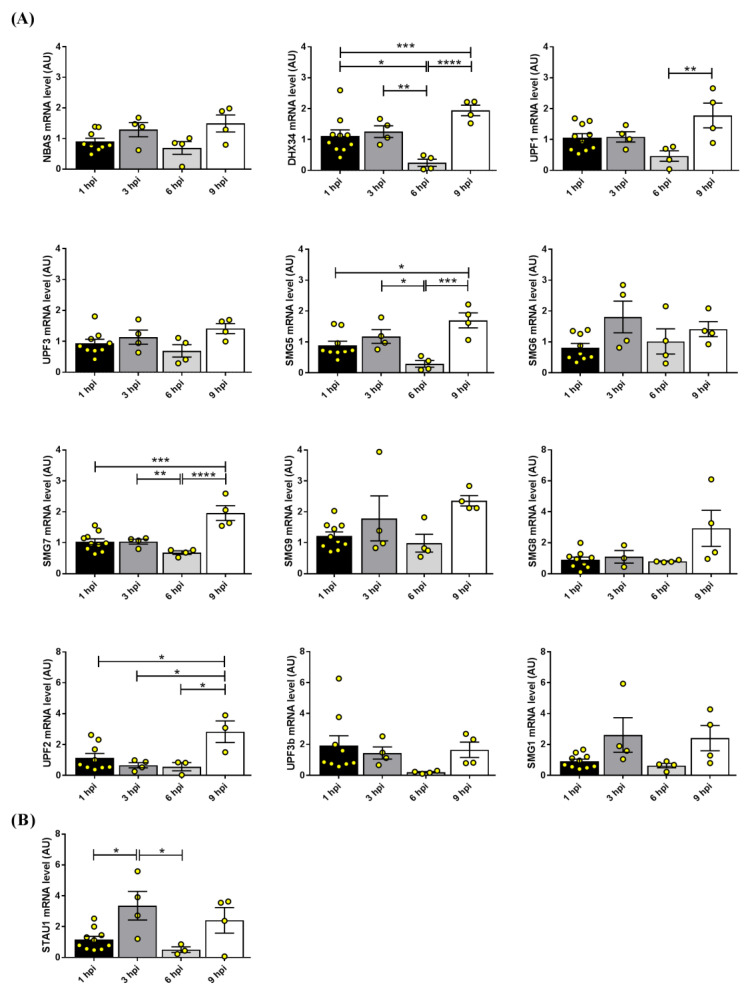
Gene expression analysis of *NBAS* and core genes of the NMD pathway (**A**) and *STAU1* (**B**) during SARS-CoV-2 infection of Calu3 cells. Each bar represents the mean of at least four technical replicates ± standard error of the mean (SEM); analysis of the outliers was performed using the ROUT method and Q = 1%. Infected cells were compared with non-infected Calu3 cells for each time point. * *p* < 0.05, ** *p* < 0.01, *** *p* < 0.001, **** *p* < 0.0001. One-way ANOVA with the Bonferroni multiple comparison test.

In previous work, Finkel and colleagues demonstrated that the levels of the majority of cellular RNAs were reduced during a SARS-CoV-2 infection in a short timeframe (within 8 h post-infection) [24]. In a similar timeframe, we found that *DHX34* was significantly decreased at 6 h post-infection as compared to that at earlier time points (1 and 3 h post-infection), while at 9 h after infection it was significantly higher compared to that at 1 and 6 h after infection. Similarly, *SMG5* and *SMG7* were significantly decreased at 6 compared to that at 3 h after infection, while at 9 h post-infection, they were significantly higher than at 1 and 6 h. *UPF1* expression was significantly higher at 9 than that at 6 h post-infection, while *UPF2* was significantly upregulated at 9 h post-infection compared to that at all the previous timepoints (Figure 3A). *UPF3b* and *SMG9* showed a trend (respectively, a decrease at 6 h and an increase at 9 h post-infection) that did not reach a significant difference. Finally, *NBAS*, *UPF3* (alias *UPF3a*), *SMG1*, *SMG6*, and *SMG9* were not modulated.

UPF1 and UPF2 have been shown to participate also in Staufen-mediated mRNA decay, which is a degradation process mediated by the double-stranded RNA binding protein Staufen, targeting mRNAs containing inter- and intramolecular RNA duplexes within their 3′-UTR [25]. Based on their sharing of key factors, nonsense-mediated and Staufen-mediated mRNA decay are mutually exclusive mechanisms [26]. We assessed whether *STAU1* expression was affected in SARS-CoV-2 infected Calu3 cells expression and found a peak at 3 h post-infection, thus preceding *UPF1* and *UPF2* upregulation (Figure 3B). This might indicate a crosstalk and coordination between the two mRNA-control mechanisms.

#### 2.2.2. NMD Targets

**Figure 4:**
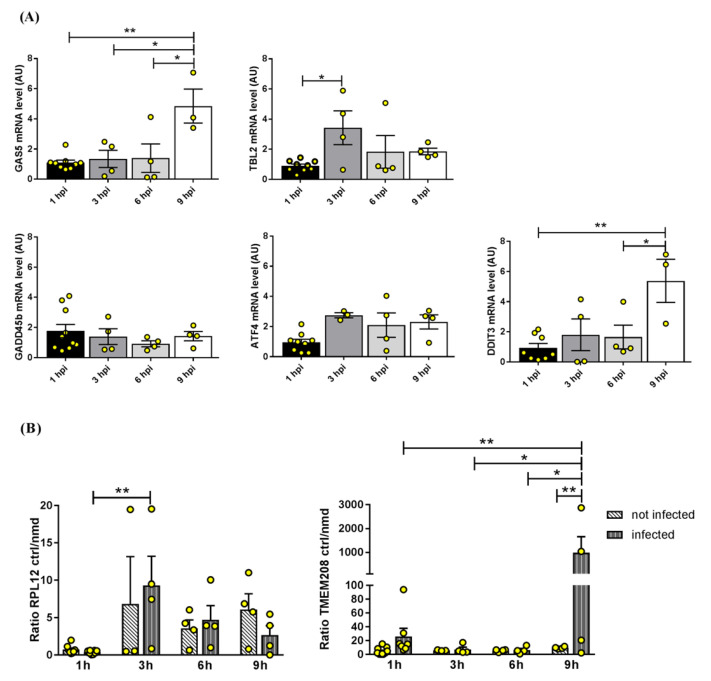
(**A**) Gene expression analysis of known targets of the NMD pathway during a SARS-CoV-2 infection of Calu3 cells. (**B**) Ratio of the abundance of NMD-non-targeted (control, ctrl) and NMD-targeted transcripts for *RPL12* and *TMEM208* genes in non-infected and SARS-CoV-2 infected Calu3 cells over time. Each bar represents the mean of at least four technical replicates ± standard error of the mean (SEM); analysis of the outliers was performed using the ROUT method and Q = 1%. * *p* < 0.05, ** *p* < 0.01. One-way or two-way ANOVA with the Bonferroni multiple comparison test, respectively.

As above described, gene expression of diverse NMD factors was mildly perturbed upon SARS-CoV-2 infection, thus we asked whether this might impact NMD function and reflect in a different abundance of substrates of this pathway [27]. To verify this hypothesis, we quantified the expression of known NMD substrates during infection. Specifically, we tested the noncoding RNA *GAS5* (Growth Arrest Specific 5), mediating growth arrest induced by several mechanisms, including NMD inhibition [28]; *TBL2* (transducin beta-like 2), coding for a PERK-binding protein involved in the ER stress response leading to upregulation of specific transcripts such as activating transcription factor 4 (*ATF4*) [29]; *GADD45B* (growth arrest and DNA damage-inducible beta), a stress sensor rapidly induced in pathophysiological stress conditions associated with growth arrest and apoptosis [30]; and *ATF4* [28,31] (Figure 4A). We found that *GAS5* and *TBL2* were significantly upregulated, respectively, at 9 and 3 h post-infection compared to the previous time points. *GADD45B* was stable over time, and *ATF4* had minor changes starting at 3 h post-infection. Interestingly, the expression of the *DDIT3* (DNA damage-inducible transcript 3) gene, coding for the C/EBP homologous protein (CHOP), which is involved in the unfolded protein response downstream to ATF4 [32], was significantly higher at 9 h post-infection compared to the previous time points.

Overall, this gene expression tuning might be ascribed to changes in the NMD pathway. On the other hand, it might also originate from unrelated effects elicited by the viral infection, such as stress responses. To investigate NMD function further, we selected two representative genes (*RPL12*, ribosomal protein L12, and *TMEM208*, transmembrane protein 208) with two diverse isoforms, i.e., a protein-producing transcript and an NMD-sensitive transcript [33,34]. In this case, changes in the ratio between the two isoforms may serve as an indicator of alteration in the NMD, as reported in the literature [33,34]. We quantified each isoform by qPCR in infected and not infected Calu3 cells in the same timeframe as above and calculated the ratio between the two isoforms at the different time points. We found that the control (protein-producing)/NMD-sensitive transcript ratio was overall stable over time for both genes in non-infected cells, while it increased in favor of the control isoform (not targeted by NMD) at 3 and 9 h after infection for *RPL12* and *TMEM208*, respectively (Figure 4B). Moreover, the control/NMD-sensitive transcript ratio for *TMEM208* at 9 h post-infection was also significantly higher in infected versus non-infected cells. Taken together with the expression pattern observed for some NMD factors (i.e., *DHX34*, *UPF1*, *UPF2*, *SMG5*, and *SMG7*), this might be interpreted as an activation of the NMD pathway early after a SARS-CoV-2 infection of Calu3 cells [35]. In this light, increased *GAS5*, *TBL2*, *ATF4*, and *DDIT3* would be explained as related to cell stress upon infection.

#### 2.2.3. Components of the Syntaxin 18 Complex

**Figure 5:**
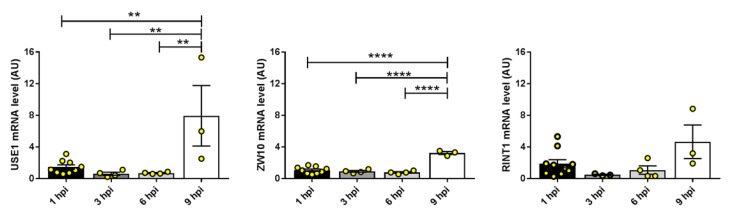
Gene expression analysis of components of the syntaxin 18 complex known to interact with NBAS during SARS-CoV-2 infection of Calu3 cells. Each bar represents the mean of at least four technical replicates ± standard error of the mean (SEM); analysis of the outliers was performed using the ROUT method and Q = 1%. ** *p* < 0.01, **** *p* < 0.0001. One-way ANOVA with the Bonferroni multiple comparison test.

As mentioned, NBAS is a part of the syntaxin 18 complex, which comprises also the SNARE proteins BNIP1 (BCL2 interacting protein 1), p31 (*alias* USE1, unconventional SNARE in the ER 1), and Sec22b and the peripheral membrane components Sly1 (*alias* SCFD1, Sec1 family domain-containing protein 1), ZW10 (Zeste White 10 homolog) and RINT-1 (RAD50 interactor 1); altogether this complex carries out cargo trafficking from the ER to the Golgi. NBAS interacts specifically with USE1, ZW10, and RINT1 [14], so we assessed the gene expression of these components in our experimental set up. We found that *USE1* and *ZW10* expression was significantly increased in infected Calu3 cells at 9 h post-infection compared to the previous time points, and *RINT1* had a similar trend (Figure 5).

Over a longer period of time, overall the expression level of most of the genes studied here did not change within 72 hours of infection (Figure S1). Based on this, protein levels were not quantified. In fact, we reasoned that putative changes of the amount of protein in the narrow time window where gene modulation was observed (i.e., up to 9 h after infection) could have been hardly ascribed to an effect of viral infection on gene expression but rather to some direct effect of the virus on the protein not tested here. A noticeable exception was represented by *GAS5* and *GADD45b,* whose expression levels increased overtime, pointing to growing cell stress during the course of infection, likely initiating cell apoptosis at the later time point of this analysis.

### 2.3. Immunofluorescence Analysis in SARS-CoV-2-Infected Calu3 Cells

**Figure 6:**
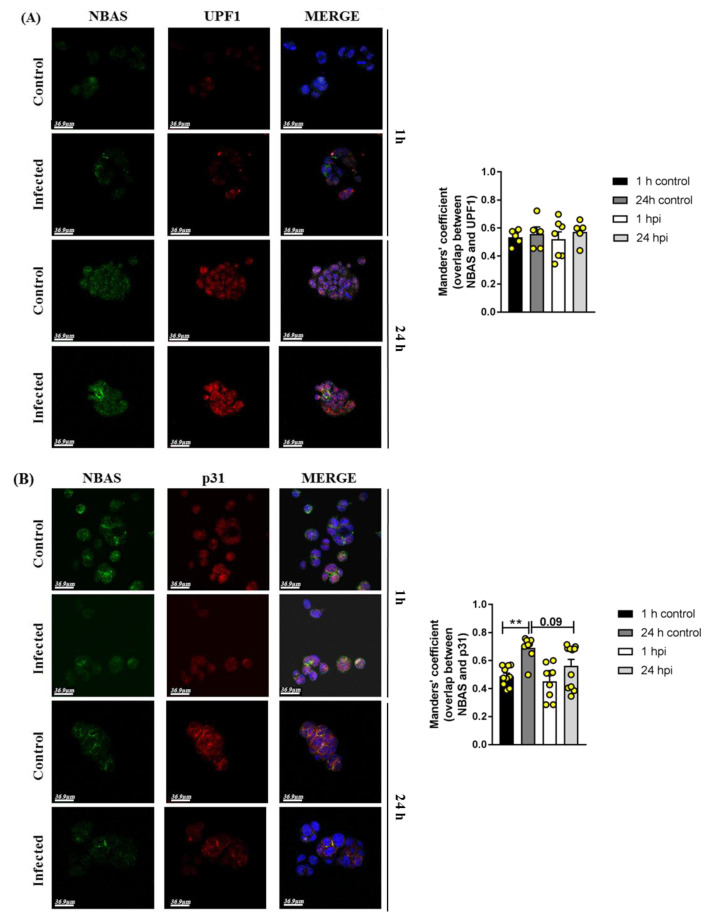
Representative images of immunofluorescence analysis in infected and non-infected (control) Calu3 cells stained at 1 and 24 h post-infection (hpi) for NBAS and UPF1 (**A**) and for NBAS and p31 (**B**); nuclei are shown in blue (DAPI). Scale bar: 36.9 µm. Manders’ coefficient was calculated to quantify co-localization of NBAS and UPF1, and NBAS and p31 proteins. ** *p* < 0.01. One-way ANOVA with the Bonferroni multiple comparison test.

Extensive organelle reshaping has been described upon SARS-CoV-2 infection [7]; this might result in altered protein interaction and function. Based on this, we conducted double immunofluorescence analysis in infected versus non-infected Calu3 cells to assess co-localization of the NBAS protein with its functional partners UPF1 and p31 (USE1, the component of the syntaxin 18 complex directly interacting with NBAS) [14]. Co-localization analysis showed that in the short timeframe, neither NBAS and UPF1 (Figure 6A), nor NBAS and p31 co-localization (Figure 6B) was affected by SARS-CoV-2 infection.

**Figure 7:**
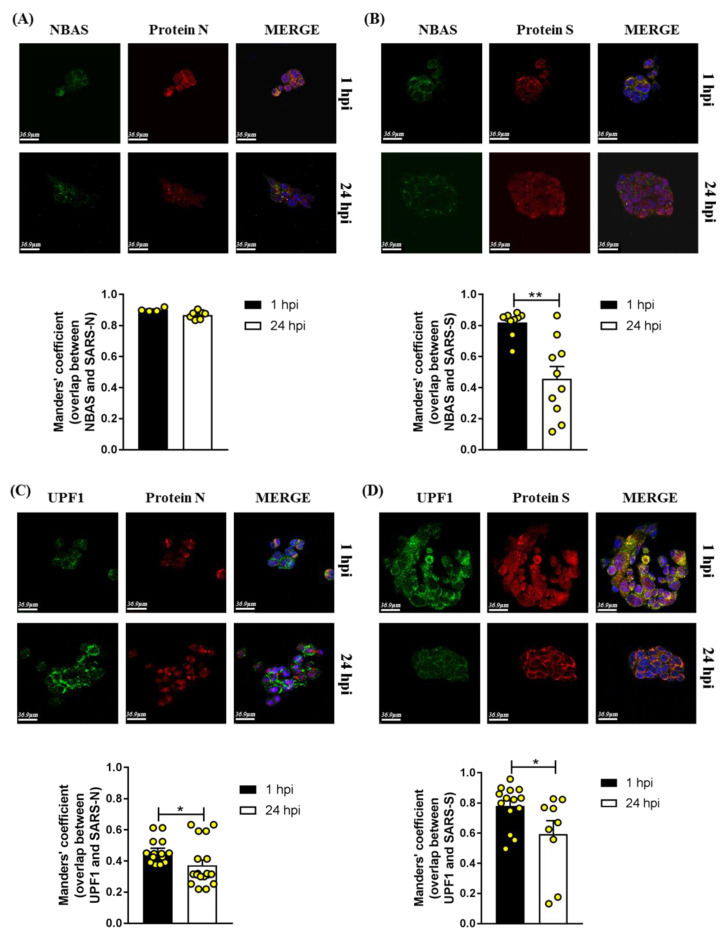
Representative images of immunofluorescence analysis of infected Calu3 cells stained at 1 and 24 h post-infection (hpi) for NBAS, UPF1, and SARS-CoV-2 proteins (N or spike); nuclei are shown in blue (DAPI). Scale bar: 36.9 µm. Manders’ coefficient was calculated for the quantification of the co-localization of NBAS or UPF1 proteins with protein N (panels (**A**,**C**), respectively) or with spike protein (panels (**B**,**D**), respectively) * *p* < 0.05, ** *p* < 0.01, Mann–Whitney test.

The SARS-CoV-2 N protein has been predicted to interact with UPF1 [11], while no data are available in the literature regarding a possible interaction between SARS-CoV-2 proteins and NBAS. In our experimental setting, we found that NBAS co-localized with N and S SARS-CoV-2 proteins (Figure 7A,B), and that while the former was stable over 24 h post-infection (Figure 7A), the latter decreased at 24 vs. 1 h after infection (Figure 7B). UPF1 co-localized with N and S SARS-CoV-2 proteins, too (Figure 7C,D), and in both cases, the co-localization decreased at 24 vs. 1 h after infection. This reduction at the latter time point might be due to ongoing assembly and release of viral particles.

## 3. Discussion

Since the COVID-19 outbreak, much effort has been given (understandably) to studying the mechanism of cell entry and the virion structure, looking for potential surface epitopes relevant for induction of neutralizing the immune response. Less attention has been paid (at least in the first pandemic wave) to elucidating how SARS-CoV-2 alters intracellular pathways. For example, as an RNA virus, SARS-CoV-2 must interface with numerous aspects of the RNA biology of the host cell, such as RNA processing, transport, translation, stability, and quality control. Host factors engaged in these mechanisms may turn out to be potentially pro-viral and constitute attractive targets for antiviral therapy, since they are genetically more stable than viral targets and often shared among related viruses [8].

The NMD pathway is a cellular function also relevant to virus biology. In fact, many viruses, comprising also SARS-CoV-2, owing to their compact genome structures, produce RNAs with atypical features, namely multiple open reading frames with internal termination codons creating a long 3′-untranslated region that could predispose them to recognition by NMD machinery. It has been previously shown that the betacoronavirus murine hepatitis virus mRNAs were indeed targeted by the NMD pathway and that, in turn, murine hepatitis virus replication induced NMD inhibition specifically through the protective role of the viral structural protein N [4].

In our work, we investigated NMD function in Calu3 cells in the early phases after SARS-CoV-2 infection, with a focus on NBAS, which acts together with UPF1 in an NMD response associated with the ER [36]. Data from the literature suggest a possible role of NBAS at the interface between the host and pathogens based on the clinical evidence of increased susceptibility to pulmonary infections in individuals bearing biallelic pathogenic variants in this gene [18]. Of note, impaired NK cell cytotoxicity and degranulation have recently been demonstrated in patients carrying an NBAS biallelic variant and in an NBAS-deficient NK cell line, owing to dysregulation of lytic vesicle transport [20]. We found that upon SARS-CoV-2 infection in Calu3 cells, NBAS expression had minor fluctuation and that also other NMD genes tested had limited gene expression changes in our experimental context. This might have several not mutually exclusive explanations. First, the close connection of NMD with many different cellular pathways and their dynamic interplay to face a viral infection [28]; moreover, the presence of diverse levels of regulation of the NMD function [37]; finally, the limited cytopathic effect of SARS-CoV-2 on Calu3 cells, which, in fact, are more tolerant as compared to other cell lines [38]. On the other hand, it is noticeable that in the literature, depletion of UPF1 or SMG5 or SMG7 has been associated with increased susceptibility to infection by positive-strand RNA viruses [39] and that, accordingly, in our experimental setting, downregulation of *SMG5* and *SMG7* occurred concomitantly with an exponential increase of viral genes.

We exploited the expression of some NMD target genes (namely, *ATF4*, *TBL2*, *GAS5*, and *GADD45B*) to infer NMD activity. These genes are associated with ER stress (*ATF4* and *TBL2*), cell stress (*GAS5*), and apoptosis (*GADD45B*). Their increased expression in infected Calu3 cells was in line with NMD involvement in the integrated stress response and with progressive cellular distress as an infection develops [40]. In addition, we assessed the ratio of mRNAs derived from the NMD-free allele to the NMD-inducing one for two genes (*RPL12* and *TMEM208*) that have diverse transcript isoforms. The ratio peaked at different time points after infection for the two genes and might suggest activation of NMD during the infection. This hypothesis should be verified extending the analysis to a larger number of genes, since NMD-targeting efficiency varies among genes and conditions.

As a cellular quality control mechanism, NMD coordinates and shares factors (UPF1 in primes) with other mechanisms, such as Staufen-mediated decay [25,26]. In our experimental setting, the key factor *STAU1* showed three-fold increased expression in infected versus non-infected cells at 3 h post-infection. Whether this translates to a functional effect should be formally assessed. Interestingly, recent protein structural analyses of SARS-CoV-2 proteins have shown that the viral helicase nsp13 potentially mimics UPF1 bound to UPF2, pointing to a viral strategy to interfere not only with NMD but also with other cellular functions featuring these factors [41].

In general, our results are in line with the data in the literature demonstrating that the SARS-CoV-2 infection modulates gene expression in Calu3 cells at short time points, with different kinetics and trends: a substantial reduction occurred for most transcripts, while other genes (particularly those related to the immune response) showed early or late upregulation or transient induction. In our work, the lack of a clear trend of expression for various genes along the infection could be due in part to the likely inherent variability of a mixed cell population. In fact, a previous study of SARS-CoV-2 infection kinetics in Calu3 cells showed that the percentage of infected cells exceeded 60% of the total at 24 h post-infection, underlining the heterogeneity of the cell population at earlier time points [42]. Moreover, a broad range of cell-to-cell variability of NMD efficiency has been recently demonstrated in a study addressing this aspect at the single-cell level [43]; the same might occur as well in our experimental setting.

The NBAS protein is involved in an additional important cellular mechanism, i.e., Golgi-to-ER retrograde transport [14]. This is one of the intracellular membrane pathways generally exploited by viruses [44]; in the case of coronaviruses, COP I vesicles have been implicated in directing the coronavirus S protein to the ERGIC near the viral assembly sites [45]. Here, we showed that in the first 24 h after SARS-CoV-2 infection, NBAS and p31’s relative localization was not altered, possibly indicating maintained vesicular trafficking in this short interval, as needed for viral particle assembly. In addition, preserved NBAS-UPF1 co-localization in infected versus non-infected cells might suggest that NMD function is conserved in the first 24 h after infection, at least for what pertains to the ER-targeted mechanism. On the other hand, the co-localization of NBAS and UPF1 with N and S proteins might indicate the possibility of viral piracy of the mechanisms implying these factors.

Recent work demonstrates extensive reshaping of intracellular membranes and organelles, comprising Golgi fragmentation and ER contribution to the viral replication complex, in SARS-CoV-2 infected lung cells [46,47]. We may speculate that intracellular localization (and function as well) of the molecules herein investigated is affected by these mechanisms and should be analyzed with more powerful technologies such as immunogold electron microscopy. The data regarding expression levels of *ZW10* and *RINT1* as members (together with NBAS) of the tethering complex involved in the recruitment of ERGIC membranes to the ER [15] should be interpreted in this framework, too. Therefore, deeper investigation about NBAS and related factors and pathways in pathophysiological conditions would give a better understanding of these biological mechanisms.

Finally, biallelic pathogenic variants in the NBAS gene have been recently associated with the presence of dysfunctional natural killer cells and impaired adaptive humoral immunity in patients [18]. This knowledge adds support to our study and confirms that NBAS is a host factor deserving further investigation from diverse perspectives in the framework of a SARS-CoV-2 infection.

## 4. Materials and Methods

### 4.1. Cells and Viral Isolate

The human lung epithelial cell line Calu3 was cultured in a complete medium composed of Dulbecco’s modified Eagle’s medium (DMEM) supplemented with 10% fetal bovine serum (FBS, Euroclone, Pero, Italy), 2 mM L-glutamine (Euroclone, Pero, Italy), and 1% penicillin/streptomycin (Euroclone, Pero, Italy). The cells were kept in a humidified 5% CO_2_ incubator at 37 °C and expanded every 3 days at a ratio of 1:3 until use. The SARS-CoV-2 isolate used to carry out the experiments (GISAID accession ID: EPI_ISL_413489) was kindly provided by Prof. Nicasio Mancini (IRCCS San Raffaele Scientific Institute, Milan, Italy). It was obtained in March 2020 from a female SARS-CoV-2-infected individual and stored until use at −80 °C. This viral isolate carried the D614G mutation in the spike protein, thus it differed from the original Wuhan strain. Trained personnel performed all infection experiments with the SARS-CoV-2 isolate exclusively at the Biosafety Level 3 (BLS-3) laboratory of IRCCS San Raffaele Scientific Institute [48], according to WHO guidelines.

### 4.2. Viral Infection

Calu3 cells were plated at 1 × 10^5^ cell/well in 48-well plates in a complete medium. Twenty-four hours later, SARS-CoV-2 was added at the multiplicity of infection of 3. The virus was adsorbed for 1 h, then the inoculum was removed, and complete medium was added. Supernatants and RNA were collected at specific time points post-infection and stored at −80 °C until use. The kinetics of viral replication were determined by a plaque assay of the viral supernatants in Vero cells, whereas both genomic and subgenomic viral RNAs were quantified in the RNA extracted from the infected cells.

### 4.3. Plaque-Forming Unit Assay

To determine the viral titers of the supernatants collected from Calu3 cells, confluent Vero cells (2.5 × 10^5^ cell/well) were seeded in 24-well plates (Corning, New York, NY, USA) 24 h prior to infection. Then, the cells were incubated with 300 µL of EMEM supplemented with 1% FBS containing serially diluted virus-containing supernatants. After 1 h of incubation, viral inoculi were removed, and the cells were covered with 500 µL of 1% methylcellulose (Sigma-Aldrich, Missouri, USA) dissolved in EMEM supplemented with 1% FBS. Three days post-infection, the cells were fixed with 4% paraformaldehyde and stained with 1% crystal violet (Sigma-Aldrich, Missouri, USA) in 70% methanol. The plaques were counted after examination with a stereoscopic microscope (SMZ-1500; Nikon Instruments Amstelveen, The Netherlands), and the virus titer was calculated in terms of plaque-forming units/mL.

### 4.4. Real-Time PCR

Total cellular RNA was isolated from SARS-CoV-2 infected and non-infected Calu3 cells at specific time points by using a TRIzol Plus RNA purification kit, followed by DNase I treatment (Invitrogen, Waltham, MA, USA), and reverse-transcribed through the SuperScript first-strand synthesis system (Invitrogen, Waltham, MA, USA) with random hexamers, according to the manufacturer’s instructions. A 72-bp fragment specific to the transcript of the nucleocapsid gene (N) was used to detect and quantify SARS-CoV-2 genomic transcripts in the infected cells with the following primer pair: N-for 5′-GACCCCAAAATCAGCGAAAT-3′ and N-rev 5′-TCTGGTTACTGCCAGTTGAATCTG-3′. The viral subgenomic RNAs were detected by a primer pair that specifically amplifies a fragment that includes the leader sequence (L/N-for-5′-AACCAACTTTCGATCTCTTGTAGATCT-3′) and the nucleocapsid gene (L/N-rev-5-CCATTCTGGTTACTGCCAGTTGAA-3′). Quantitative RT-PCR was performed on a VIIA7 or ABI 7500 Prism instrument (Applied Biosystems, Waltham, MA, USA) using Universal SybrGreen supermix (Bio-Rad, Hercules, CA, USA), following the manufacturer’s protocol. The 18S or GAPDH genes were used as housekeeping genes. All the cellular genes analyzed are listed in Table 1. For each candidate, gene expression relative to the housekeeping gene was conducted following the comparative 2^−ΔΔCt^ method, and normalized expression was calculated as the relative mRNA level (arbitrary units, AU) compared to that in the non-infected controls.

### 4.5. Coimmunofluorescence Analysis

First, 1 × 10^5^ Calu3 cells were seeded on polylysine-coated cover slips (diameter 15 mm) placed at the bottom of the wells of 24-well plates. Twenty-four hours later, the cells were infected as described above and fixed at 1 or 24 h post-infection with 4% paraformaldehyde (PFA; Sigma Aldrich, St. Louis, MO, USA) for 15 min at room temperature. Then, the cells were permeabilized with 0.3% Triton™ X-100 (Sigma Aldrich, St. Louis, MO, USA) for 10 min in PBS and blocked with PBS 5% FBS for 1 h at room temperature. Primary antibodies (anti-NBAS Atlas Antibodies, 1:500; anti-UPF1 Atlas Antibodies, 1:250; anti-USE1 (p31), Sigma 1:250; anti-Protein N Invitrogen, 1:250; anti-spike protein, Proteintech, 1:250) were used following the manufacturer’s instructions and as previously published [16,17,49]. The primary antibodies were diluted in a blocking buffer and incubated for 2 h, at room temperature. Appropriate secondary antibodies conjugated with Alexa Fluor 488 or 594 were incubated for 1 h at room temperature. Regarding NBAS/UPF1 and NBAS/p31 co-immunostaining, since these antibodies were raised against the same species (rabbit), the cells were first stained for NBAS (followed by its secondary antibody), then blocked with PBS 5% FBS as described above, and finally stained for either UPF1 or p31 (followed by its secondary antibody). On the other hand, for coimmunostaining of NBAS or UPF1 and the N or S viral proteins, the primary antibodies could be applied to the fixed cells simultaneously; then, similarly, the respective secondary antibodies were added together after washing. The slides were mounted with the VECTASHIELD Antifade Mounting Medium with DAPI (Vector Laboratories).

Images were acquired with an inverted SP8I confocal microscope (LeicaMicrosystems, Buccinasco, Italy), using the Leica acquisition software. Lasers and spectra detection bands were selected for optimal imaging of secondary antibodies. Z-stack of 25 optical sections with thickness of about 0.3–0.4 µm and with 63× magnification was performed for each condition, capturing 5 different fields. Two-channel co-localization analysis was conducted using ImageJ software (64 bit Java 8); Manders’ correlation coefficient was calculated through the Coloc 2 plugin. The analysis was conducted considering a ROI on each single cell for all the images to evaluate the overlap between channel 1 (in red) and channel 2 (in green).

### 4.6. Statistical Analysis

Data were analyzed using GraphPad Prism 7 and represented as average values. For each graph, the identification of outliers with the ROUT method and Q = 1% was performed. In gene expression and co-localization analyses, the results from treated and untreated cells were compared with one-way ANOVA with Bonferroni multiple comparison test; for post hoc comparison of co-localization, we considered 1 h infected versus 1 h control, 1 h infected versus 24 h infected, 1 h control versus 24 h control, and 24 h infected versus 24 h control. Two-way ANOVA with a Bonferroni multiple comparison test was used to calculate p-values for co-localization in transfected cells without infection. Significance was represented as follows: * *p* < 0.05, ** *p* < 0.01, *** *p* < 0.001, **** *p* < 0.0001.

## Figures and Tables

**Table 1:** Primers used for qRT-PCR analysis.

Genes	Forward Primer Sequences (5′-3′)	Reverse Primer Sequences (5′-3′)
**NBAS**	GCAGTACAAGAGGATGAAGTAGG	AGGACTTTCATGGTGGTAGC
**DHX34**	TGGGATGGACACAAGTTCATT	GACCGGAAGTTAAGGCGTTC
**UPF1**	CAACGAGCACCAAGGCATT	ATGACGCCATACCTTGCTCT
**UPF2**	AATTCTCAATCCTTAGCAGACCT	CAGGCCCATGTTCTTCTGGT
**UPF3**	CCCGGTGCAGTCGTAAAA	TTATCACTGCCGCTGTGTG
**UPF3B**	AAGAAGCGCTGAGCAAGGT	CATGCTCAGGCATAGGTTGA
**SMG1**	GCACCTGAAGTAGCCAAATCT	TTCTCCCTGACTGGCATTGT
**SMG5**	CCAGGCACAGTTCCGAAT	ATGTCTCTCATGAGCCTGTTCC
**SMG6**	CGGGATCCTGGCTACTCTG	CCTGGCCTCCTTTAATTCCT
**SMG7**	GAAAGCAGAATGTGGCAGTG	TGGGGTTTGAGTTACAGGTGTT
**SMG8**	CCATCAGCTCTGTGAGGAGA	AGCACAGGCGGATTTCTATC
**SMG9**	TTGCACCATGGGAAAGAGA	GAGGTGGTGGCTGTTTTGAC
**ATF4**	TCTCCAGCGACAAGGCTAA	CCAATCTGTCCCGGAGAA
**GAS5**	GCATTAGACAGAAACTGGAAGT	CATGGATAAAAACGTTACCAGGA
**GADD45B**	CATTGTCTCCTGGTCACGAA	TAGGGGACCCACTGGTTGT
**TBL2**	TTCAGCCCTGACTGCAGAG	TTGAAGACACGGAGGGTGT
**STAU1**	TGCCAAAGCGTTGAGGAT	TCTTCTTCGGATTCTCTTCCAT
**IL6**	AAATTCGGTACATCCTCGACG	GGAAGGTTCAGGTTGTTTTCTGC
**DDIT3**	TGGAAGCCTGGTATGAGGAC	TGTGACCTCTGCTGGTTCTG
**18S**	CGCAGCTAGGAATAATGGAATAGG	CATGGCCTCAGTTCCGAAA
**IFN-B**	CCAACAAGTGTCTCCTCCAAATT	GTAGGAATCCAAGCAAGTTGTAGCT
**GAPDH**	CCACCCATGGCAAATTCC	TGGGATTTCCATTGATGACAAG
**USE1**	GAAGGACAACCAGACCCTGTC	GACGCTCTGACTCCGTCTTC
**RINT1**	CAGTGCCCCGGAGATATACA	TGCTTTGGCTCAGTAAGTAATTCA
**ZW10**	TGTTGTACCAACATATCACAAGGA	CATACAGTTGTTGTGATGAATAGCAG
**RPL12-CTRL**	GTGCAACTTCCTTCGGTCGT	CGTTGCCTTGGCAATGTCAT
**RPL12-NMD**	GAGGACTGGACCACCTGTGG	CGTTGCCTTGGCAATGTCAT
**TMEM208-CTRL**	GTTGGCCCTGGGCTTTAGTC	GAGAAGCAGCTGAGCACCTG
**TMEM208-NMD**	GTTGGCCCTGGGCTTTAGTC	CGGTGGGGGACACTCA

## Data Availability

Data supporting the reported results can be found at https://zenodo.org/communities/humanitasirccs and will be made available upon request to the corresponding authors.

## Supplementary Materials

The following supporting information can be downloaded at: https://www.mdpi.com/article/10.3390/ijms24032634/s1.
