# Supplementary material for: Modulation of NBAS-Related Functions in the Early Response to SARS-CoV-2 Infection
Source: Int J Mol Sci. 2023 Jan 30;24(3):2634. doi: 10.3390/ijms24032634 (PMC9916797; doi:10.3390/ijms24032634)
Supplement: Supplementary file 1 [file ijms-24-02634-s001.zip › ijms-2134940-supplementary.pdf]

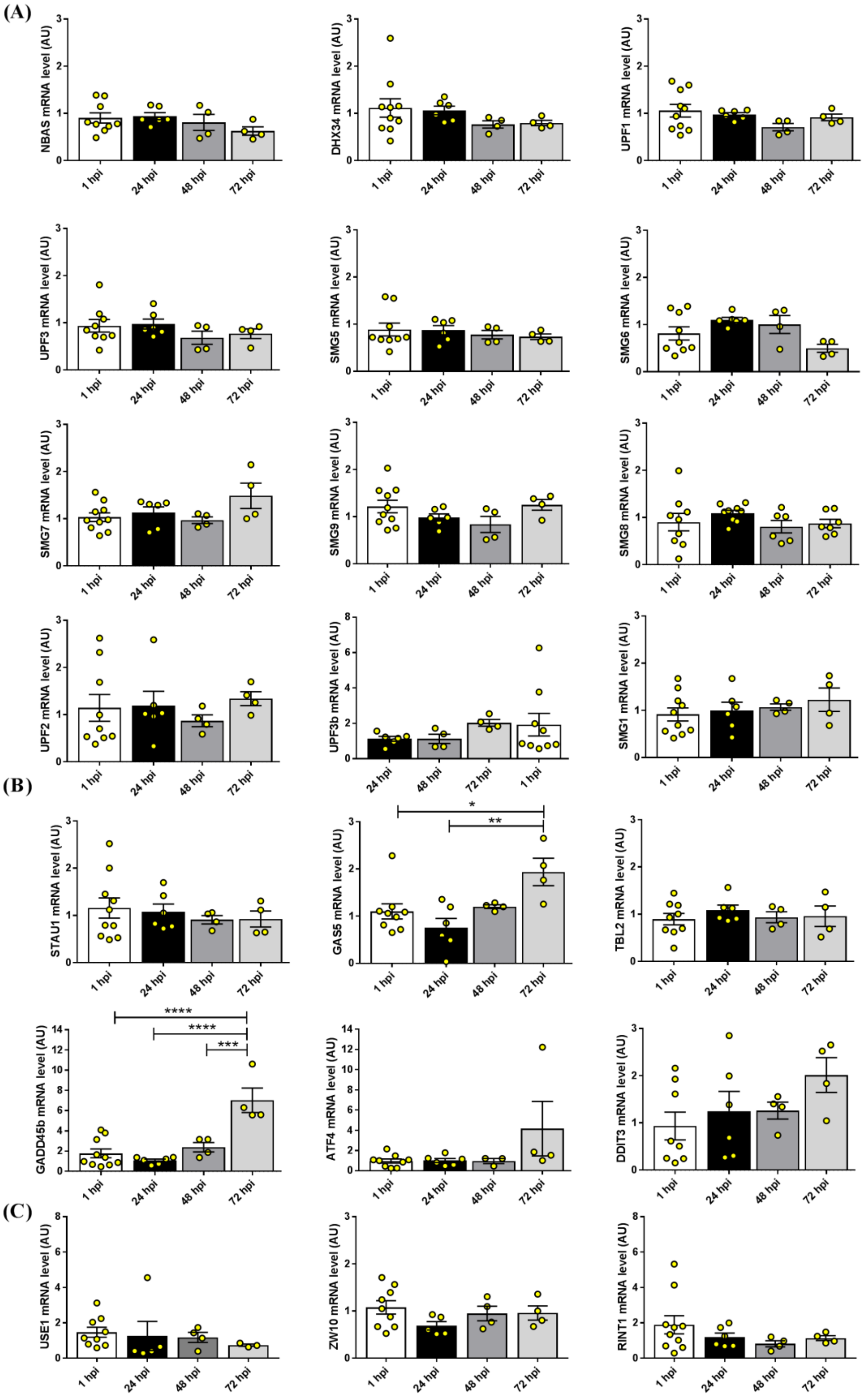

Figure S1. Gene expression analysis in SARS-CoV-2 infected vs not-infected Calu3 cells up to 72 hours-post-infection (hpi). (**A**) *NBAS* and core genes of the NMD pathway: (**B**) *STAU1* and known NMD targets; and (**C**) components of the syntaxin 18 complex known to interact with NBAS. Each bar represents the mean of at least four technical replicates ± standard error of the mean (SEM); analysis of the outliers was performed using the ROUT method and Q =1%. * $p < 0.05$, ** $p < 0.01$, *** $p < 0.001$, **** $p < 0.0001$. One-way ANOVA with the Bonferroni multiple comparison test.
